# Synthesis, crystal structure and Hirshfeld surface analysis of the tetra­kis complex NaNdPyr_4_(*i*-PrOH)_2_·*i*-PrOH with a carbacyl­amido­phosphate of the amide type

**DOI:** 10.1107/S2056989023010071

**Published:** 2023-11-30

**Authors:** Nataliia S. Kariaka, Viktoriya V. Dyakonenko, Kateryna O. Znovjyak, Svitlana V. Shishkina, Volodymyr M. Amirkhanov

**Affiliations:** a Department of Chemistry, Kyiv National Taras Shevchenko University, Volodymyrska str. 64, 01601 Kyiv, Ukraine; b SSI "Institute for Single Crystals", National Academy of Sciences of Ukraine, Nauky ave. 60, 61001 Kharkiv, Ukraine; Tulane University, USA

**Keywords:** crystal structure, carbacyl­amido­phosphate, neodymium, tetra­kis-complex

## Abstract

The tetra­kis complex of neodymium(III), tetra­kis­{μ-*N*-[bis­(pyrrolidin-1-yl)phos­phor­yl]acet­am­id­ato}bis(pro­pan-2-ol)neodymiumsodium pro­pan-2-ol monosol­vate, [NaNd(C_10_H_16_Cl_3_N_3_O_2_)_4_(C_3_H_8_O)_2_]·C_3_H_8_O or NaNdPyr_4_(*i*-PrOH)_2_·*i*-PrOH, with the amide type CAPh ligand bis(*N*,*N*-tetra­methylene)(tri­chloro­acetyl)phos­phoric acid tri­amide (HPyr), has been synthesized, crystallized and characterized by X-ray diffraction. The complex does not have the tetra­kis­(CAPh)lanthanide anion, which is typical for ester-type CAPh-based coordin­ation compounds. Instead, the NdO_8_ polyhedron is formed by one oxygen atom of a 2-propanol mol­ecule and seven oxygen atoms of CAPh ligands in the title compound. Three CAPh ligands are coordinated in a bidentate chelating manner to the Nd^III^ ion and simultaneously binding the sodium cation by μ_2_-bridging PO and CO groups while the fourth CAPh ligand is coordinated to the sodium cation in a bidentate chelating manner and, due to the μ_2_-bridging function of the PO group, also binds the neodymium ion.

## Abstract 2

The crystal structure of neodymium tetra­kis complex based on bis(*N*,*N*-tetra­methylene)(tri­chloro­acetyl)phos­phoric acid tri­amide is reported and discussed.

## 1. Chemical context

Carbacyl­amido­phosphates (CAPh, H*L*) belong to an attractive class of organic compounds due to their biological activity (Grimes *et al.*, 2008 ▸; Grynyuk *et al.*, 2016 ▸; Oroujzadeh *et al.*, 2017 ▸; Amirkhanov *et al.*, 2019 ▸), ability to bind metals and create complexes with biological or pharmacological activity (Dorosti *et al.*, 2019 ▸) as well as highly luminescent lanthanides complexes (Kariaka *et al.*, 2018 ▸; Pham *et al.*, 2020*a* ▸).

Among CAPh-based luminescent lanthanide compounds, tetra­kis-complexes, (cation)[*LnL*_4_], are of special inter­est because of the full saturation of the lanthanide coordination sphere with the formation of an *Ln*O_8_ polyhedron that shields the metal from the quenching effects of the solvent mol­ecules. To date, CAPh-based lanthanide tetra­kis-complexes are known only for the ester-type CAPhs (*i.e.* CAPhs with ester-type substituents at the phospho­rus atom) with no structures of tetra­kis-complexes with amide-type CAPhs (*i.e.* CAPhs with amide-type substituents at the phospho­rus atom) reported (Amirkhanov *et al.*, 2014 ▸). Aiming to synthesize the tetra­kis-complex with an amide-type CAPh [bis(*N*,*N*-tetra­methylene)(tri­chloro­acetyl)phos­phoric acid tri­amide (HPyr)], the title compound of formula NaNdPyr_4_(*i*-PrOH)_2_·*i*-PrOH was obtained. Herein the synthesis and crystal structure, including characterization of the inter­molecular contacts by Hirshfeld surface analysis, of NaNdPyr_4_(*i*-PrOH)_2_·*i*-PrOH are presented. 
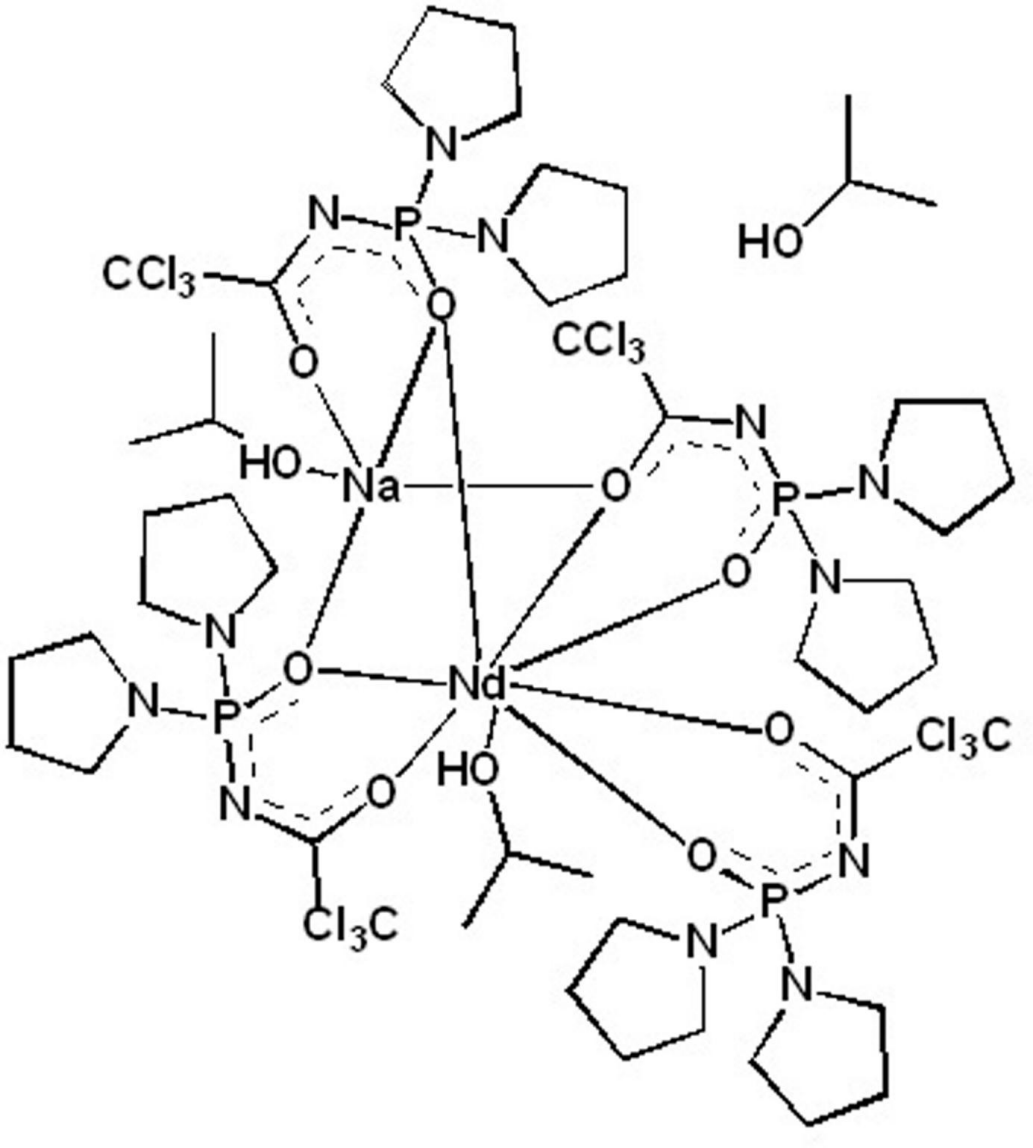


## 2. Structural commentary

**Figure 1:**
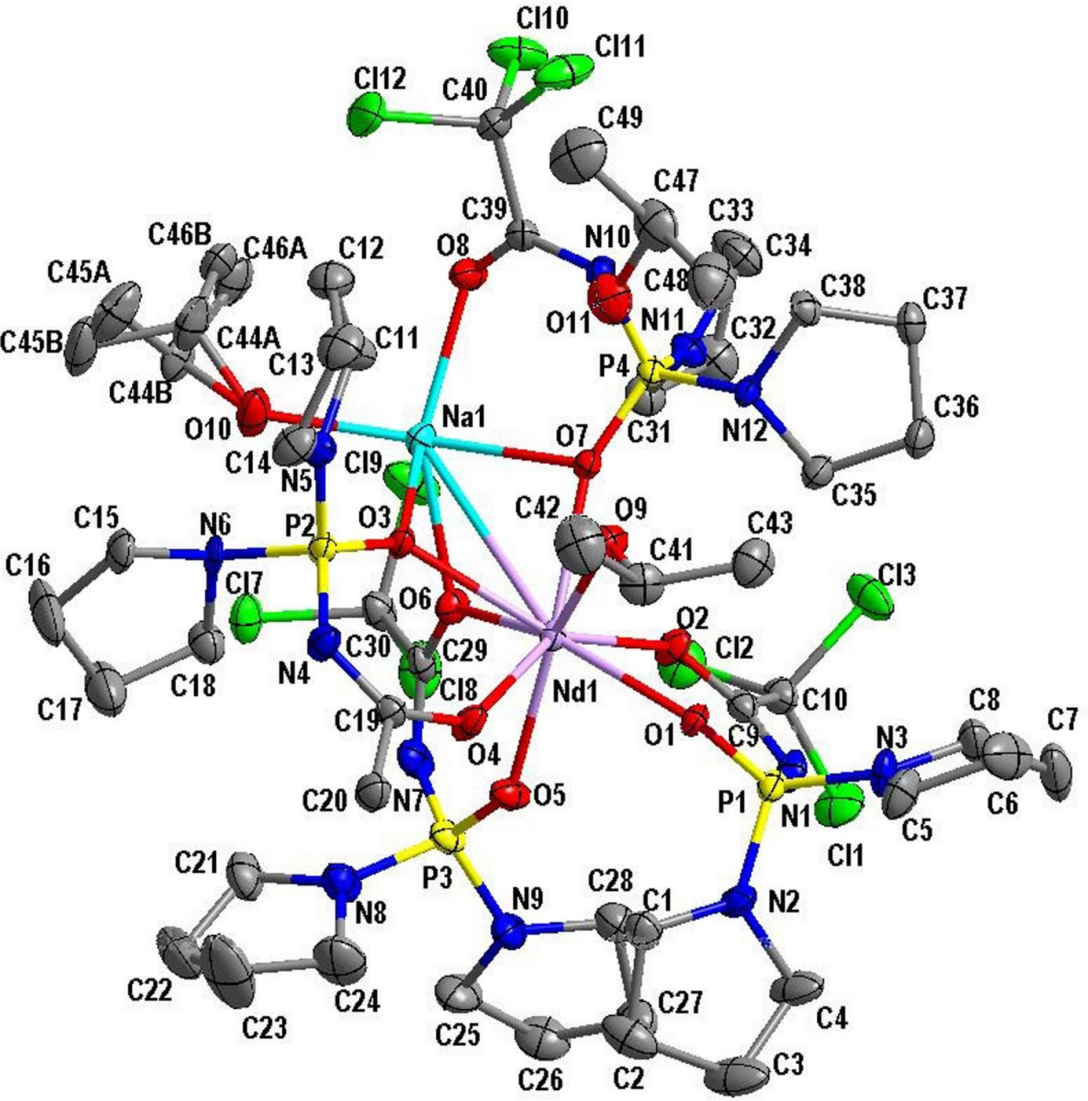
The mol­ecular structure of the title compound with displacement ellipsoids drawn at the 50% probability level. All the hydrogen atoms and disordered chlorine atoms are omitted for clarity.

The title compound crystallizes in the triclinic crystal system with two mol­ecules in the unit cell. The mol­ecular structure of the title compound is shown in Fig. 1 ▸.

The neodymium atom has coordination number eight; however, unlike typical CAPh-based tetra­kis-complexes, the NdO_8_ polyhedron is formed by seven oxygen atoms of CAPh ligands and one oxygen atom of a 2-propanol mol­ecule. All of the four CAPh anions are involved in binding the neodymium ion, but each of them in a different mode. One of the CAPhs is coordinated to the neodymium cation in the typical bidentate chelating mode while two others are coordinated to the neodymium ion in the bidentate chelating mode and additionally, due to the μ_2_-bridging function of the PO or CO group, are coordinated to the sodium cation as well. The fourth CAPh ligand is coordinated to the sodium cation in a bidentate chelating manner and, due to μ_2_-bridging function of the PO group, is coordinated to the neodymium ion as well. The coordination polyhedron of Nd^III^ can be inter­preted with the *SHAPE2.1* program (Llunell *et al.*, 2013 ▸) as a square anti­prism (*D*_4*d*_) (Table 1 ▸). The sodium cation polyhedron, NaO_5_Cl, can be inter­preted as a trigonal prism (*D*_3*h*_). The coordination environment of the sodium cation consists of five oxygen atoms and one chlorine atom. The two oxygen atoms are from CAPh ligands coordinated to sodium in a bidentate chelating mode, one more oxygen is from the μ_2_-bridging PO group of the other CAPh, the chlorine atom and one more oxygen atom are from a bridging CAPh in which the CO group has the μ_2-_bridging function, and the fifth oxygen is from a 2-propanol mol­ecule.

Selected bonds lengths for the title compound are given in Table 2 ▸. The Nd—O(P) bonds are shorter than the Nd—O(C) bonds. Among the Nd—O(P) bonds, the longest is that for the μ_2_-bridging oxygen atom (Nd1—O3). Among the Nd—O(C) bonds, the longest is also that for the μ_2_-bridging oxygen atom (Nd1—O6). The neodymium–oxygen bond to the 2-propanol mol­ecule (Nd1—O9) is longer than the average values for the Nd—O(P) and Nd—O(C) bonds. All the Nd—O bonds are shorter than the sum of van der Waals radii of oxygen and the Nd^3+^ ionic radius (2.61 Å). For the sodium cation, the Na—O bond lengths follow the trend *d[*Na—O(*i*-PrOH)] < *d*[Na—O(C)] < *d*[Na—O(P)]. The Na—O(P) bonds are longer than the sum of the O^2−^ and Na^+^ ionic radii (2.37 Å) but shorter, however, than the sum of Na^+^ ionic radius and oxygen’s van der Waals radius (2.52 Å). The Na1—Cl9 bond is also longer than the sum of the Na^+^ ionic radius and chlorine’s van der Waals radius (2.75 Å), which points to the ionic character of this bond. The Na1—Cl9 bond length [3.0192 (19) Å] is comparable to reported Na⋯Cl inter­actions in CAPh-based complexes (2.98–3.22 Å; Amirkhanov *et al.*, 1996 ▸; Trush *et al.*, 2005 ▸). Compared to HPyr (Gholivand *et al.*, 2006 ▸), the C—O and P—O bonds are longer and the P—N and C—N bonds are shorter in the title compound. The bond lengths of the μ_2_-bridging P—O and C—O groups are comparable to those in the C—O and P—O groups that are coordinated to one metal. Thus, the μ_2_-bridging function does not influence the C—O and P—O bond lengths.

In the title compound, an intra­molecular hydrogen bond is observed between the hydrogen atom H10 of the 2-propanol mol­ecule coordinated to the sodium cation and the N6 nitro­gen atom of the pyrrolidine substituent of the CAPh ligand (Table 3 ▸). The participation of the N6 atom as a proton acceptor in hydrogen bonding results in its pyramidalization (the sum of bond angles centered at the N6 atom is 340°). Another hydrogen bond exists between the hydrogen atom H11 of the solvate 2-propanol mol­ecule and the N10 nitro­gen atom of the chelating fragment of the CAPh ligand, coordinated to the sodium cation in the bidentate chelating mode (Table 3 ▸). Additionally to the hydrogen bonds, an intra­molecular contact C49—H49*A*⋯Cl11 contact is observed (Table 3 ▸).

## 3. Supra­molecular features

Numerous Cl⋯Cl, Cl⋯H and H⋯H inter­molecular contacts are observed in the crystal of the title compound. The CCl_3_ and pyrrolidine substituents of the CAPh ligand as well as the 2-propanol mol­ecules participate in these contacts. The main Cl⋯Cl and Cl⋯H inter­molecular inter­actions are given in Table 4 ▸. The Cl8⋯Cl6*B* inter­actions, at 3.04 Å, and Cl2⋯Cl6*A* inter­actions, at 3.42 Å, are less than the sum of the chlorine atoms van der Waals radii (3.5 Å) and are in the middle of the range (2.75–4.0 Å) reported for Cl⋯Cl inter­actions (Capdevila-Cortada *et al.*, 2016 ▸). The [θ_1_ - θ_2_] value equals 3.4° and 39.3° for the Cl8⋯Cl6*B* and Cl2⋯Cl6*A* inter­actions, respectively. Thus the first inter­action can be assigned as Type I and the latter as Type II. Among the Cl⋯H contacts, the closest are Cl5*B*—H4*A* inter­actions (2.52 Å).

## 4. Hirshfeld surface analysis and fingerprint plots

**Figure 2:**
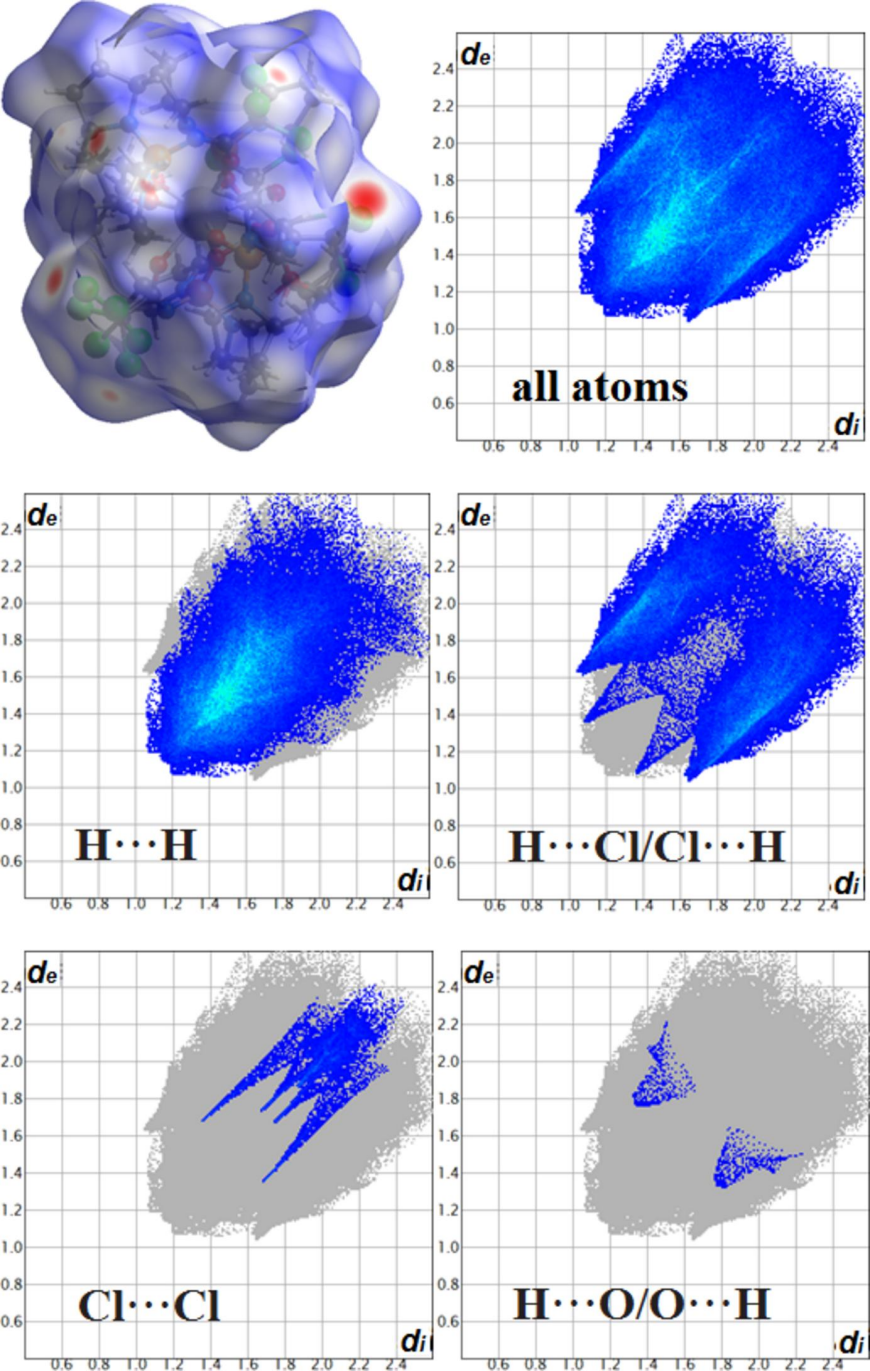
The Hirshfeld surface mapped over *d*_norm_ and two-dimensional fingerprint plots for the H⋯H (58.2%), H⋯Cl/Cl⋯H (37.4%), Cl⋯Cl (4.0%) and H⋯O/O⋯H (0.4%) inter­actions of the title compound.

The inter­molecular inter­actions in the crystal structure of the title compound were visualized with a Hirshfeld surface analysis (Fig. 2 ▸) and the corresponding two-dimensional fingerprint plots (Spackman *et al.*, 2009 ▸) using the *CrystalExplorer17* program (Turner *et al.*, 2017 ▸). The strongest contacts, which are visualized on the Hirshfeld surface as dark-red spots, correspond to the Cl⋯Cl inter­actions. The lighter red spots correspond to H⋯Cl/Cl⋯H and H⋯H contacts. The majority of the inter­molecular inter­actions of the title compound are weak, which results in the blue colour of the Hirshfeld surface. According to the fingerprint plots, the H⋯H contacts make the largest contribution to the Hirshfeld surface (58.2%) with the shortest at *d*_i_ + *d*_e_ = 2.3 Å. The second largest contribution (37.4%) belongs to H⋯Cl/Cl⋯H contacts with the shortest at *d*_i_ + *d*_e_ = 2.5 Å. The Cl⋯Cl inter­actions are not numerous and contribute only 4.0% to the surface with the shortest at *d*_i_ + *d*_e_ = 3.0 Å. The H⋯O/O⋯H inter­actions make a 0.4% contribution to the Hirshfeld surface and represent hydrogen bonds to the carbonyl group oxygen atom O8 from the hydrogen atoms of the 2-propanol mol­ecules.

## 5. Database survey

A search of the Cambridge Structural Database (CSD, Version 5.44, updated to June 2023; Groom *et al.*, 2016 ▸) found 23 structures where a metal is coordinated by four CAPh ligands. The five tetra­kis-complexes crystallize with two mol­ecules in the unit cell. Three of the complexes are binuclear, containing bis-carbacyl­amido­phosphate ligands. There are two complexes of La^III^, five of Nd^III^, one of Sm^III^, six of Eu^III^, three of Gd^III^, two of Tb^III^, one of Dy^III^ one of Er^III^, and two of Yb^III^. Most often the coordination polyhedra of the central ions in the tetra­kis-complexes are distorted square anti­prisms (*D*_4*d*_). Six cases of triangular dodeca­hedral (*D*_2*d*_) *Ln* ^III^ ion coordination polyhedra have been reported for CAPh-based tetra­kis-complexes. In the neodymium compounds, the central ions have coordination polyhedra in the form of distorted square anti­prisms (*D*_4*d*_) and the Nd—O bonds lengths are in the range 2.303–2.516 Å (Kariaka *et al.*, 2016 ▸, 2022 ▸; Pham *et al.*, 2020*b* ▸; Horniichuk *et al.*, 2021 ▸). Eleven of the reported CAPh-based tetra­kis-complexes of lanthanides contain a sodium cation as the counter-ion. All these sodium-containing complexes contain solvent mol­ecules in their lattices, while the other twelve known tetra­kis-complexes of lanthanides are solvent free. The sodium cations are six- or seven-coordinated in these complexes, being bonded to solvents, chelating core substituents of the CAPh ligands, and by bridging CO and PO groups of the chelating CAPh ligands.

## 6. Synthesis and crystallization

To obtain the complex NaNdPyr_4_(*i*-PrOH)_2_·*i*-PrOH, 0.1 mmol (0.03587 mg) of NdCl_3_·6H_2_O was dissolved in 2-propanol in the presence of the dehydrating agent HC(OC_2_H_5_)_3_ (0.6 mmol, 0.1 ml) by boiling this mixture for several minutes. This solution was added to a solution of NaPyr (0.4 mmol, 0.14826 g) in acetone. The resulting mixture was boiled for a minute then cooled to room temperature and left to stand tightly corked for a day for precipitation of NaCl. The clear solution was deca­nted and left to stand for slow evaporation of the solvent. In a few days, crystals of the target complex appeared. The crystals were filtered off, washed with cold iso­propanol and dried in air. The crystals are soluble in DMSO, methanol, acetone, aceto­nitrile and insoluble in water. IR (KBr): *ν*_max_ = 3379*w*,*br*, 2967*m*, 2868*m* [ν(CH)], 1614*s* [ν(CO)], 1460*w*, 1336*s* [ν(CN)], 1240*w*, 1205*m*, 1127*s* [(PO)], 1010*m*, 989*w* [ν(PN)], 949*w*, 910*w*, 865*m*, 813*m*, 761*w*, 673*m*, 583*m*, 526*m*, 440*w* cm^−1^.

## 7. Refinement

Crystal data, data collection and structure refinement details are summarized in Table 5 ▸. The C-bound H atoms were placed in calculated positions and refined using the riding model with x*U*_eq_(C, O), where *x* = 1.5 for hydroxyl groups and 1.2 for all other H atoms.

The structure exhibits disorder of the Cl atoms of one CCl_3_ substituent. All Cl—C bond distances were restrained to be similar to each other (within a standard deviation of 0.002 Å) and with a target value of 1.76 Å. The *U*_ij_ values of the disordered chlorine atoms were restrained to be similar to each other (within a standard deviation of 0.02 Å^2^). The disorder ratio was refined and is 0.757 (3):0.243 (3).

One of the coordinated isopropyl groups is disordered over two positions. The C—O and C—C bond distances of the two components were restrained to be equal with an effective standard deviation 0.005 Å and the *U*_ij_ values of the disordered C atoms were restrained to be similar to each other (within a standard deviation of 0.02 Å^2^). The disorder ratio was refined and is 0.529 (13):0.471 (13).

## Supplementary Material

Crystal structure: contains datablock(s) I. DOI: 10.1107/S2056989023010071/mw2199sup1.cif

Structure factors: contains datablock(s) I. DOI: 10.1107/S2056989023010071/mw2199Isup2.hkl

CCDC reference: 2309129

Additional supporting information:  crystallographic information; 3D view; checkCIF report

## Figures and Tables

**Table 1:** Continuous shape measures values for Nd1 and Na1 in the title compound OP-8 is an octa­gon, *D*_8*h*_; HPY-8 is a hepta­gonal pyramid, *C*_7*v*_; HBPY-8 is a hexa­gonal bipyramid, *D*_6*h*_; CU-8 is a cube, *O*_*h*_; SAPR-8 is a square anti­prism, *D*_4*d*_; TDD-8 is a triangular dodeca­hedron, *D*_2*d*_; JGBF-8 is a Johnson-gyrobifastigium (J26), *D*_2*d*_; JETBPY-8 is a Johnson-elongated triangular bipyramid (J14), *D*_3*h*_; JBTP-8 is a Johnson-biaugmented trigonal prism (J50), *C*_2*v*_; BTPR-8 is a biaugmented trigonal prism, *C*_2*v*_; JSD-8 is a snub disphenoid (J84), *D*_2*d*_; TT-8 is a triakis tetra­hedron, *T_d_*; ETBPY-8 is an elongated trigonal bipyramid, *D*_3*h*_.

Nd1	OP-8	HPY-8	HBPY-8	CU-8	SAPR-8	TDD-8	ETBPY-8
	29.441	22.884	15.960	9.477	0.388	2.318	14.685
	JGBF-8	JETBPY-8	JBTPR-8	BTPR-8	JSD-8	TT-8	-
	27.709	2.164	1.935	4.197	10.282	23.994	-
Na1	Hexagon (*D*_6*h*_)	Penta­gonal pyramid (*C*_5*v*_)	Octa­hedron (O_*h*_)	Trigonal prism (*D*_3*h*_)	Johnson penta­gonal pyramid J2 (*C*_5*v*_)	-	-
	33.334	18.031	9.098	4.986	22.006	-	-

**Table 2:** Selected bond lengths (Å)

Nd1—O1	2.364 (3)	P4—O7	1.505 (3)
Nd1—O2	2.422 (3)	P4—N10	1.633 (3)
Nd1—O3	2.431 (3)	Na1—O3	2.407 (3)
Nd1—O4	2.478 (2)	Na1—O6	2.369 (3)
Nd1—O5	2.366 (3)	Na1—O7	2.418 (3)
Nd1—O6	2.573 (2)	Na1—O8	2.296 (3)
Nd1—O7	2.413 (3)	Na1—O10	2.275 (3)
Nd1—O9	2.543 (3)	O2—C9	1.248 (5)
Cl9—Na1	3.0192 (19)	O4—C19	1.255 (5)
P1—O1	1.501 (3)	O6—C29	1.257 (5)
P1—N1	1.630 (3)	O8—C39	1.240 (4)
P2—O3	1.500 (3)	N1—C9	1.298 (5)
P2—N4	1.622 (3)	N4—C19	1.288 (5)
P3—O5	1.500 (3)	N7—C29	1.292 (5)
P3—N7	1.625 (3)	N10—C39	1.325 (5)

**Table 3:** Hydrogen-bond geometry (Å, °)

*D*—H⋯*A*	*D*—H	H⋯*A*	*D*⋯*A*	*D*—H⋯*A*
O10—H10*A*⋯N6	0.85	2.11	2.872 (4)	148
O11—H11⋯N10	0.84	2.22	3.024 (6)	160
C49—H49*A*⋯Cl11	0.98	2.82	3.637 (7)	141

**Table 4:** Inter­molecular Cl⋯Cl and Cl⋯H inter­actions in the title compound (Å)

Atom 1	Atom 2	Symmetry atom 1	Symmetry atom 2	Contact distance
Cl6*A*	C12	*x*, *y*, *z*	−1 + *x*, *y*, *z*	3.419
Cl6*B*	Cl8	*x*, *y*, *z*	*x*, *y*, *z*	3.038
Cl1	H3*A*	*x*, *y*, *z*	2 − *x*, 1 − *y*, 1 − *z*	2.750
Cl4*A*	H7*B*	*x*, *y*, *z*	−1 + *x*, −1 + *y*, *z*	2.770
Cl5*A*	H24*A*	*x*, *y*, *z*	*x*, *y*, *z*	2.913
Cl5*A*	H5*A*	*x*, *y*, *z*	1 − *x*, 1 − *y*, 1 − *z*	2.830
Cl5*A*	H6*A*	*x*, *y*, *z*	1 − *x*, 1 − *y*, 1 − *z*	2.943
Cl5*B*	H4*A*	*x*, *y*, *z*	*x*, *y*, *z*	2.517
Cl6*A*	H24*C*	*x*, *y*, *z*	*x*, *y*, *z*	2.903
Cl7	H48*C*	*x*, *y*, *z*	*x*, −1 + *y*, *z*	2.878
C18	H37*A*	*x*, *y*, *z*	*x*, −1 + *y*, *z*	2.943
Cl9	H31*A*	*x*, *y*, *z*	*x*, *y*, *z*	2.835
Cl11	H38*B*	*x*, *y*, *z*	1 − *x*, 1 − *y*, −*z*	2.936
Cl11	H49*A*	*x*, *y*, *z*	*x*, *y*, *z*	2.823
Cl12	H12*B*	*x*, *y*, *z*	−*x*, −*y*, −*z*	2.874

**Table 5:** Experimental details

Crystal data	
Chemical formula	[NaNd(C_10_H_16_Cl_3_N_3_O_2_)_4_(C_3_H_8_O)_2_]·C_3_H_8_O
*M*_r_	1737.82
Crystal system, space group	Triclinic, *P* 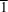
Temperature (K)	103
*a*, *b*, *c* (Å)	12.7712 (4), 13.6102 (4), 24.7288 (8)
α, β, γ (°)	98.301 (3), 97.814 (3), 117.051 (3)
*V* (Å^3^)	3687.5 (2)
*Z*	2
Radiation type	Mo *K*α
μ (mm^−1^)	1.29
Crystal size (mm)	0.5 × 0.3 × 0.2

Data collection	
Diffractometer	Xcalibur, Sapphire3
Absorption correction	Multi-scan (*CrysAlis PRO*, Agilent, 2014 ▸)
*T*_min_, *T*_max_	0.711, 1.000
No. of measured, independent and observed [*I* > 2σ(*I*)] reflections	29184, 14464, 12181
*R*_int_	0.047
(sin θ/λ)_max_ (Å^−1^)	0.617

Refinement	
*R*[*F* ^2^ > 2σ(*F* ^2^)], *wR*(*F* ^2^), *S*	0.047, 0.110, 1.04
No. of reflections	14464
No. of parameters	877
No. of restraints	87
H-atom treatment	H-atom parameters constrained
Δρ_max_, Δρ_min_ (e Å^−3^)	1.76, −1.34

Computer programs: *CrysAlis PRO* (Agilent, 2014 ▸), *OLEX2.solve* (Bourhis *et al.*, 2015 ▸), *SHELXL2019/3* (Sheldrick, 2015 ▸) and *OLEX2* (Dolomanov *et al.*, 2009 ▸).

## supplementary crystallographic information

### Crystal data

**Table:**

[NaNd(C_10_H_16_Cl_3_N_3_O_2_)_4_(C_3_H_8_O)_2_]·C_3_H_8_O	*Z* = 2
*M**_r_* = 1737.82	*F*(000) = 1778
Triclinic, *P*1	*D*_x_ = 1.565 Mg m^−^^3^
*a* = 12.7712 (4) Å	Mo *K*α radiation, λ = 0.71073 Å
*b* = 13.6102 (4) Å	Cell parameters from 6677 reflections
*c* = 24.7288 (8) Å	θ = 3.3–30.1°
α = 98.301 (3)°	µ = 1.29 mm^−^^1^
β = 97.814 (3)°	*T* = 103 K
γ = 117.051 (3)°	Block, colourless
*V* = 3687.5 (2) Å^3^	0.5 × 0.3 × 0.2 mm

### Data collection

**Table:**

Xcalibur, Sapphire3 diffractometer	12181 reflections with *I* > 2σ(*I*)
Detector resolution: 16.1827 pixels mm^-1^	*R*_int_ = 0.047
ω scans	θ_max_ = 26.0°, θ_min_ = 3.1°
Absorption correction: multi-scan (CrysAlisPro, Agilent, 2014)	*h* = −15→15
*T*_min_ = 0.711, *T*_max_ = 1.000	*k* = −16→16
29184 measured reflections	*l* = −29→30
14464 independent reflections	

### Refinement

**Table:**

Refinement on *F*^2^	Primary atom site location: iterative
Least-squares matrix: full	Hydrogen site location: mixed
*R*[*F*^2^ > 2σ(*F*^2^)] = 0.047	H-atom parameters constrained
*wR*(*F*^2^) = 0.110	*w* = 1/[σ^2^(*F*_o_^2^) + (0.0411*P*)^2^ + 3.2978*P*] where *P* = (*F*_o_^2^ + 2*F*_c_^2^)/3
*S* = 1.04	(Δ/σ)_max_ = 0.002
14464 reflections	Δρ_max_ = 1.76 e Å^−^^3^
877 parameters	Δρ_min_ = −1.34 e Å^−^^3^
87 restraints	

### Special details

**Table:**

Geometry. All esds (except the esd in the dihedral angle between two l.s. planes) are estimated using the full covariance matrix. The cell esds are taken into account individually in the estimation of esds in distances, angles and torsion angles; correlations between esds in cell parameters are only used when they are defined by crystal symmetry. An approximate (isotropic) treatment of cell esds is used for estimating esds involving l.s. planes.

### Fractional atomic coordinates and isotropic or equivalent isotropic displacement parameters (Å^2^)

**Table:**

	*x*	*y*	*z*	*U*_iso_*/*U*_eq_	Occ. (<1)
Nd1	0.49634 (2)	0.27520 (2)	0.27799 (2)	0.01594 (7)	
Cl1	1.03087 (10)	0.48921 (11)	0.38922 (5)	0.0403 (3)	
Cl2	0.90782 (10)	0.32199 (10)	0.28514 (5)	0.0360 (3)	
Cl3	0.98196 (10)	0.55767 (10)	0.28839 (6)	0.0421 (3)	
Cl4A	0.0187 (3)	−0.00580 (19)	0.34464 (11)	0.1293 (17)	0.757 (3)
Cl4B	0.1629 (9)	0.0463 (9)	0.3960 (4)	0.094 (2)	0.243 (3)
Cl5A	0.23064 (17)	0.1698 (3)	0.42076 (7)	0.0729 (8)	0.757 (3)
Cl5B	0.1912 (9)	0.2662 (4)	0.3961 (4)	0.103 (3)	0.243 (3)
Cl6A	0.0784 (3)	0.2221 (3)	0.35197 (12)	0.0916 (10)	0.757 (3)
Cl6B	−0.0108 (3)	0.0943 (7)	0.3317 (2)	0.0723 (19)	0.243 (3)
Cl7	0.51289 (10)	−0.14415 (8)	0.19092 (5)	0.0329 (3)	
Cl8	0.76740 (10)	0.01020 (9)	0.23771 (6)	0.0391 (3)	
Cl9	0.64096 (13)	0.04819 (10)	0.14551 (6)	0.0455 (3)	
Cl10	0.47945 (12)	0.26494 (11)	−0.04654 (5)	0.0404 (3)	
Cl11	0.34069 (13)	0.36827 (11)	−0.01198 (5)	0.0450 (3)	
Cl12	0.24487 (13)	0.12831 (11)	−0.02834 (6)	0.0577 (4)	
P1	0.68858 (9)	0.50325 (8)	0.39560 (5)	0.0199 (2)	
P2	0.19201 (9)	0.06061 (8)	0.20288 (4)	0.0197 (2)	
P3	0.57031 (10)	0.09822 (9)	0.35435 (5)	0.0237 (2)	
P4	0.56916 (9)	0.39976 (8)	0.15111 (4)	0.0170 (2)	
Na1	0.45014 (14)	0.12135 (12)	0.14265 (7)	0.0228 (3)	
O1	0.5723 (2)	0.4383 (2)	0.35177 (11)	0.0190 (6)	
O2	0.7142 (2)	0.3698 (2)	0.29281 (12)	0.0237 (6)	
O3	0.3248 (2)	0.1274 (2)	0.20604 (11)	0.0197 (6)	
O4	0.3203 (2)	0.2102 (2)	0.32181 (12)	0.0249 (6)	
O5	0.5434 (3)	0.1933 (2)	0.34924 (12)	0.0266 (7)	
O6	0.5470 (2)	0.1249 (2)	0.23225 (12)	0.0223 (6)	
O7	0.5357 (2)	0.3189 (2)	0.18929 (11)	0.0179 (6)	
O8	0.4573 (3)	0.1925 (2)	0.06370 (12)	0.0275 (7)	
O9	0.3904 (3)	0.3862 (2)	0.25091 (13)	0.0268 (7)	
H9	0.397249	0.410390	0.221507	0.040*	
O10	0.2997 (3)	−0.0579 (2)	0.10109 (14)	0.0401 (8)	
H10	0.250000	−0.099104	0.118919	0.060*	0.5
H10A	0.236290	−0.071254	0.113299	0.060*	0.5
N1	0.8054 (3)	0.4951 (3)	0.38100 (16)	0.0246 (8)	
N2	0.6586 (3)	0.4606 (3)	0.45281 (15)	0.0292 (8)	
N3	0.7394 (3)	0.6402 (3)	0.41128 (16)	0.0284 (8)	
N4	0.1428 (3)	0.0721 (3)	0.25932 (15)	0.0252 (8)	
N5	0.1126 (3)	0.0863 (3)	0.15567 (15)	0.0238 (7)	
N6	0.1567 (3)	−0.0758 (2)	0.18312 (15)	0.0230 (7)	
N7	0.6067 (3)	0.0480 (3)	0.30070 (16)	0.0267 (8)	
N8	0.4565 (3)	−0.0133 (3)	0.36409 (16)	0.0310 (9)	
N9	0.6800 (3)	0.1423 (3)	0.41036 (16)	0.0279 (8)	
N10	0.4694 (3)	0.3681 (3)	0.09354 (14)	0.0189 (7)	
N11	0.6959 (3)	0.4136 (3)	0.13640 (15)	0.0226 (7)	
N12	0.5901 (3)	0.5236 (2)	0.18370 (14)	0.0190 (7)	
C1	0.5453 (4)	0.3650 (3)	0.45645 (19)	0.0286 (10)	
H1A	0.512718	0.302348	0.422533	0.034*	
H1B	0.483826	0.388929	0.461063	0.034*	
C2	0.5812 (5)	0.3299 (4)	0.5076 (2)	0.0422 (13)	
H2A	0.617529	0.280652	0.498780	0.051*	
H2B	0.511369	0.289857	0.524035	0.051*	
C3	0.6742 (5)	0.4442 (5)	0.5467 (2)	0.0559 (16)	
H3A	0.725477	0.434554	0.576997	0.067*	
H3B	0.634606	0.484722	0.563610	0.067*	
C4	0.7488 (5)	0.5077 (5)	0.5065 (2)	0.0456 (13)	
H4A	0.781452	0.590683	0.518867	0.055*	
H4B	0.816280	0.491529	0.503744	0.055*	
C5	0.6656 (4)	0.6852 (4)	0.4342 (2)	0.0374 (11)	
H5A	0.661599	0.675783	0.472949	0.045*	
H5B	0.582588	0.647205	0.410483	0.045*	
C6	0.7320 (6)	0.8091 (4)	0.4332 (2)	0.0538 (16)	
H6A	0.721021	0.856429	0.463878	0.065*	
H6B	0.703812	0.821662	0.396837	0.065*	
C7	0.8625 (6)	0.8352 (4)	0.4415 (3)	0.0573 (17)	
H7A	0.898353	0.845605	0.481406	0.069*	
H7B	0.911594	0.904113	0.428638	0.069*	
C8	0.8532 (4)	0.7300 (3)	0.4052 (2)	0.0375 (12)	
H8A	0.850356	0.735427	0.365542	0.045*	
H8B	0.921708	0.717755	0.419088	0.045*	
C9	0.7993 (4)	0.4347 (3)	0.33380 (18)	0.0219 (9)	
C10	0.9243 (4)	0.4482 (3)	0.32560 (19)	0.0267 (9)	
C11	0.1571 (4)	0.1238 (4)	0.1063 (2)	0.0342 (11)	
H11A	0.174705	0.068258	0.084552	0.041*	
H11B	0.230991	0.198797	0.117616	0.041*	
C12	0.0523 (4)	0.1304 (4)	0.0724 (2)	0.0374 (11)	
H12A	0.081805	0.190661	0.051388	0.045*	
H12B	−0.004482	0.057109	0.045631	0.045*	
C13	−0.0074 (4)	0.1582 (4)	0.1168 (2)	0.0390 (11)	
H13A	−0.092127	0.136553	0.100241	0.047*	
H13B	0.036992	0.240215	0.135058	0.047*	
C14	−0.0013 (4)	0.0879 (4)	0.1583 (2)	0.0342 (11)	
H14A	0.001232	0.124039	0.196516	0.041*	
H14B	−0.071186	0.010324	0.146772	0.041*	
C15	0.0259 (4)	−0.1618 (3)	0.1704 (2)	0.0305 (10)	
H15A	−0.012015	−0.178076	0.130164	0.037*	
H15B	−0.018091	−0.135545	0.193409	0.037*	
C16	0.0271 (5)	−0.2651 (4)	0.1854 (3)	0.0634 (19)	
H16A	−0.047638	−0.311826	0.197247	0.076*	
H16B	0.033250	−0.312115	0.152776	0.076*	
C17	0.1322 (5)	−0.2225 (4)	0.2313 (3)	0.0565 (16)	
H17A	0.163012	−0.277416	0.231042	0.068*	
H17B	0.111258	−0.209751	0.267814	0.068*	
C18	0.2263 (4)	−0.1118 (4)	0.2221 (2)	0.0310 (10)	
H18A	0.268215	−0.054103	0.258040	0.037*	
H18B	0.286902	−0.122901	0.204910	0.037*	
C19	0.2099 (4)	0.1389 (3)	0.30703 (17)	0.0199 (8)	
C20	0.1410 (2)	0.13080 (17)	0.35323 (11)	0.0283 (9)	
C21	0.4217 (4)	−0.1327 (4)	0.3399 (2)	0.0388 (12)	
H21A	0.493079	−0.144510	0.344113	0.047*	
H21B	0.381118	−0.155830	0.299668	0.047*	
C22	0.3356 (6)	−0.1982 (5)	0.3742 (3)	0.0652 (19)	
H22A	0.266356	−0.268326	0.349690	0.078*	
H22B	0.377445	−0.219144	0.403320	0.078*	
C23	0.2946 (7)	−0.1226 (5)	0.4000 (3)	0.082 (3)	
H23A	0.271867	−0.141178	0.435386	0.099*	
H23B	0.223668	−0.129639	0.374291	0.099*	
C24	0.4008 (5)	−0.0032 (5)	0.4115 (3)	0.0528 (15)	
H24A	0.373329	0.053993	0.410848	0.063*	
H24B	0.456984	0.016448	0.447939	0.063*	
C25	0.7147 (5)	0.0603 (4)	0.4286 (2)	0.0406 (12)	
H25A	0.707111	0.004495	0.395730	0.049*	
H25B	0.662880	0.019150	0.452838	0.049*	
C26	0.8459 (5)	0.1314 (4)	0.4611 (2)	0.0433 (13)	
H26A	0.858573	0.108214	0.496550	0.052*	
H26B	0.901013	0.123467	0.438473	0.052*	
C27	0.8672 (4)	0.2525 (4)	0.4730 (2)	0.0403 (12)	
H27A	0.953310	0.307546	0.476898	0.048*	
H27B	0.842143	0.270416	0.507652	0.048*	
C28	0.7882 (4)	0.2537 (4)	0.4216 (2)	0.0366 (11)	
H28A	0.768411	0.315956	0.429572	0.044*	
H28B	0.827835	0.261739	0.389525	0.044*	
C29	0.5903 (4)	0.0671 (3)	0.25138 (18)	0.0218 (9)	
C30	0.6282 (4)	0.0005 (3)	0.20847 (19)	0.0263 (9)	
C31	0.7450 (4)	0.3373 (4)	0.1459 (2)	0.0289 (10)	
H31A	0.693024	0.260688	0.121060	0.035*	
H31B	0.753474	0.331076	0.185448	0.035*	
C32	0.8665 (4)	0.3943 (4)	0.1315 (2)	0.0388 (12)	
H32A	0.895308	0.339348	0.121279	0.047*	
H32B	0.927344	0.456570	0.163255	0.047*	
C33	0.8409 (5)	0.4400 (4)	0.0814 (2)	0.0411 (13)	
H33A	0.915123	0.506164	0.078237	0.049*	
H33B	0.808890	0.380735	0.046084	0.049*	
C34	0.7471 (4)	0.4742 (3)	0.09373 (19)	0.0270 (10)	
H34A	0.785127	0.557317	0.108344	0.032*	
H34B	0.683711	0.451284	0.059416	0.032*	
C35	0.6867 (4)	0.5840 (3)	0.23585 (18)	0.0259 (9)	
H35A	0.752612	0.565127	0.234395	0.031*	
H35B	0.654178	0.563618	0.269120	0.031*	
C36	0.7320 (4)	0.7099 (3)	0.23815 (19)	0.0305 (10)	
H36A	0.819797	0.755123	0.254934	0.037*	
H36B	0.688848	0.739233	0.260213	0.037*	
C37	0.7040 (4)	0.7131 (3)	0.17651 (19)	0.0258 (9)	
H37A	0.699726	0.782744	0.172780	0.031*	
H37B	0.765168	0.708700	0.157144	0.031*	
C38	0.5816 (4)	0.6081 (3)	0.15374 (18)	0.0221 (9)	
H38A	0.516029	0.623268	0.162134	0.026*	
H38B	0.566813	0.581165	0.112639	0.026*	
C39	0.4400 (4)	0.2729 (3)	0.05720 (17)	0.0210 (8)	
C40	0.3771 (4)	0.2606 (3)	−0.00414 (18)	0.0250 (9)	
C41	0.3456 (4)	0.4442 (4)	0.2866 (2)	0.0294 (10)	
H41	0.352934	0.425105	0.324028	0.035*	
C42	0.2144 (5)	0.4031 (5)	0.2626 (3)	0.0495 (14)	
H42A	0.204399	0.416574	0.224885	0.074*	
H42B	0.185272	0.444109	0.286765	0.074*	
H42C	0.167984	0.321629	0.260605	0.074*	
C43	0.4235 (4)	0.5717 (4)	0.2952 (2)	0.0382 (12)	
H43A	0.508074	0.593482	0.310091	0.057*	
H43B	0.396821	0.611127	0.321738	0.057*	
H43C	0.415707	0.592785	0.259144	0.057*	
C44A	0.2358 (9)	−0.1163 (8)	0.0434 (3)	0.041 (3)	0.471 (13)
H44A	0.191559	−0.076784	0.029733	0.050*	0.471 (13)
C44B	0.2746 (7)	−0.1499 (6)	0.0555 (3)	0.028 (2)	0.529 (13)
H44B	0.285587	−0.211073	0.069053	0.033*	0.529 (13)
C45A	0.1449 (9)	−0.1910 (13)	0.0270 (6)	0.054 (4)	0.529 (13)
H45A	0.120681	−0.251005	−0.006701	0.081*	0.529 (13)
H45B	0.136871	−0.127674	0.016341	0.081*	0.529 (13)
H45C	0.092815	−0.220959	0.052817	0.081*	0.529 (13)
C46A	0.3608 (14)	−0.0959 (16)	0.0192 (7)	0.037 (4)	0.529 (13)
H46A	0.344239	−0.152885	−0.014593	0.055*	0.529 (13)
H46B	0.444019	−0.065902	0.040293	0.055*	0.529 (13)
H46C	0.350228	−0.033923	0.008447	0.055*	0.529 (13)
O11	0.2881 (3)	0.4343 (3)	0.12862 (16)	0.0409 (8)	
H11	0.330496	0.415945	0.110808	0.061*	
C47	0.2591 (4)	0.5094 (4)	0.1028 (2)	0.0424 (12)	
H47	0.327306	0.556729	0.086403	0.051*	
C48	0.2436 (5)	0.5860 (5)	0.1484 (3)	0.0552 (15)	
H48A	0.178250	0.539704	0.165243	0.083*	
H48B	0.223655	0.638458	0.131949	0.083*	
H48C	0.318942	0.629328	0.177307	0.083*	
C49	0.1441 (5)	0.4434 (5)	0.0567 (3)	0.0555 (15)	
H49A	0.155668	0.394957	0.027430	0.083*	
H49B	0.125343	0.496635	0.040404	0.083*	
H49C	0.077274	0.396181	0.072548	0.083*	
C46B	0.3264 (16)	−0.105 (2)	0.0081 (8)	0.038 (5)	0.471 (13)
H46D	0.373296	−0.140323	0.021351	0.057*	0.471 (13)
H46E	0.380887	−0.023989	0.011201	0.057*	0.471 (13)
H46F	0.283864	−0.141844	−0.031204	0.057*	0.471 (13)
C45B	0.1435 (15)	−0.2385 (9)	0.0375 (8)	0.061 (5)	0.471 (13)
H45D	0.105600	−0.274896	−0.002342	0.091*	0.471 (13)
H45E	0.081672	−0.241710	0.057753	0.091*	0.471 (13)
H45F	0.183376	−0.278323	0.053137	0.091*	0.471 (13)

### Atomic displacement parameters (Å^2^)

**Table:**

	*U*^11^	*U*^22^	*U*^33^	*U*^12^	*U*^13^	*U*^23^
Nd1	0.01428 (11)	0.01414 (10)	0.01690 (12)	0.00585 (8)	0.00157 (9)	0.00184 (8)
Cl1	0.0198 (6)	0.0553 (7)	0.0386 (7)	0.0159 (5)	−0.0053 (5)	0.0091 (6)
Cl2	0.0272 (6)	0.0420 (6)	0.0441 (7)	0.0220 (5)	0.0081 (5)	0.0068 (5)
Cl3	0.0254 (6)	0.0456 (7)	0.0562 (9)	0.0112 (5)	0.0144 (6)	0.0305 (6)
Cl4A	0.128 (2)	0.0747 (16)	0.0567 (15)	−0.0577 (15)	0.0675 (17)	−0.0248 (12)
Cl4B	0.099 (5)	0.178 (6)	0.087 (5)	0.106 (5)	0.063 (4)	0.092 (5)
Cl5A	0.0359 (11)	0.159 (2)	0.0184 (9)	0.0443 (13)	0.0086 (8)	0.0151 (12)
Cl5B	0.105 (5)	0.112 (5)	0.066 (4)	0.025 (4)	0.065 (4)	−0.005 (4)
Cl6A	0.130 (2)	0.158 (3)	0.0868 (19)	0.126 (2)	0.0760 (18)	0.0730 (19)
Cl6B	0.060 (3)	0.128 (5)	0.054 (4)	0.057 (3)	0.039 (3)	0.030 (3)
Cl7	0.0335 (6)	0.0206 (5)	0.0398 (7)	0.0109 (4)	0.0102 (5)	−0.0012 (4)
Cl8	0.0274 (6)	0.0334 (6)	0.0587 (8)	0.0183 (5)	0.0122 (6)	0.0022 (6)
Cl9	0.0767 (10)	0.0459 (7)	0.0428 (8)	0.0437 (7)	0.0376 (8)	0.0227 (6)
Cl10	0.0530 (8)	0.0624 (8)	0.0279 (6)	0.0406 (7)	0.0191 (6)	0.0206 (6)
Cl11	0.0690 (9)	0.0608 (8)	0.0239 (6)	0.0508 (7)	0.0000 (6)	0.0056 (6)
Cl12	0.0433 (8)	0.0463 (7)	0.0366 (8)	−0.0111 (6)	−0.0110 (6)	0.0072 (6)
P1	0.0186 (5)	0.0164 (5)	0.0199 (6)	0.0068 (4)	−0.0012 (4)	0.0012 (4)
P2	0.0166 (5)	0.0180 (5)	0.0174 (5)	0.0036 (4)	0.0034 (4)	0.0010 (4)
P3	0.0318 (6)	0.0217 (5)	0.0234 (6)	0.0167 (5)	0.0070 (5)	0.0082 (4)
P4	0.0162 (5)	0.0150 (5)	0.0175 (5)	0.0065 (4)	0.0019 (4)	0.0030 (4)
Na1	0.0279 (9)	0.0188 (7)	0.0187 (9)	0.0101 (7)	0.0037 (7)	0.0015 (6)
O1	0.0172 (14)	0.0171 (13)	0.0167 (15)	0.0067 (11)	−0.0023 (12)	−0.0013 (11)
O2	0.0134 (14)	0.0295 (15)	0.0220 (16)	0.0076 (12)	0.0010 (13)	0.0002 (12)
O3	0.0176 (14)	0.0163 (13)	0.0171 (15)	0.0031 (11)	0.0026 (12)	−0.0002 (11)
O4	0.0153 (15)	0.0294 (15)	0.0187 (16)	0.0041 (12)	0.0032 (13)	−0.0024 (12)
O5	0.0417 (18)	0.0262 (15)	0.0208 (16)	0.0228 (14)	0.0085 (14)	0.0078 (12)
O6	0.0273 (16)	0.0207 (14)	0.0215 (16)	0.0149 (12)	0.0043 (13)	0.0020 (12)
O7	0.0158 (14)	0.0157 (12)	0.0178 (15)	0.0052 (11)	0.0009 (12)	0.0025 (11)
O8	0.0418 (19)	0.0239 (15)	0.0189 (16)	0.0183 (14)	0.0052 (14)	0.0048 (12)
O9	0.0327 (17)	0.0293 (15)	0.0234 (17)	0.0206 (14)	0.0035 (14)	0.0042 (13)
O10	0.043 (2)	0.0255 (16)	0.034 (2)	0.0040 (14)	0.0151 (17)	−0.0063 (14)
N1	0.0161 (17)	0.0205 (17)	0.031 (2)	0.0076 (14)	−0.0042 (16)	0.0002 (15)
N2	0.0210 (19)	0.034 (2)	0.020 (2)	0.0055 (16)	−0.0035 (16)	0.0044 (16)
N3	0.0243 (19)	0.0148 (16)	0.035 (2)	0.0041 (14)	0.0037 (17)	−0.0054 (15)
N4	0.0178 (18)	0.0269 (18)	0.022 (2)	0.0042 (14)	0.0055 (16)	0.0017 (15)
N5	0.0175 (18)	0.0258 (18)	0.023 (2)	0.0073 (14)	0.0026 (16)	0.0056 (15)
N6	0.0137 (17)	0.0117 (15)	0.029 (2)	−0.0033 (13)	0.0005 (15)	−0.0006 (14)
N7	0.037 (2)	0.0208 (17)	0.032 (2)	0.0193 (16)	0.0134 (18)	0.0094 (16)
N8	0.038 (2)	0.0267 (19)	0.033 (2)	0.0172 (17)	0.0128 (19)	0.0098 (17)
N9	0.036 (2)	0.0217 (17)	0.028 (2)	0.0164 (16)	0.0040 (18)	0.0073 (15)
N10	0.0189 (17)	0.0182 (16)	0.0176 (18)	0.0088 (14)	−0.0004 (15)	0.0029 (13)
N11	0.0230 (18)	0.0233 (17)	0.027 (2)	0.0137 (15)	0.0081 (16)	0.0098 (15)
N12	0.0206 (17)	0.0142 (15)	0.0174 (18)	0.0077 (13)	−0.0027 (14)	−0.0007 (13)
C1	0.029 (2)	0.027 (2)	0.029 (3)	0.0114 (19)	0.011 (2)	0.0068 (19)
C2	0.039 (3)	0.057 (3)	0.053 (4)	0.032 (3)	0.025 (3)	0.033 (3)
C3	0.031 (3)	0.096 (5)	0.037 (3)	0.026 (3)	0.002 (3)	0.027 (3)
C4	0.028 (3)	0.069 (4)	0.026 (3)	0.014 (3)	−0.002 (2)	0.015 (3)
C5	0.037 (3)	0.028 (2)	0.039 (3)	0.016 (2)	0.000 (2)	−0.008 (2)
C6	0.097 (5)	0.036 (3)	0.042 (3)	0.044 (3)	0.015 (3)	0.009 (2)
C7	0.069 (4)	0.021 (2)	0.058 (4)	0.000 (2)	0.029 (3)	0.002 (2)
C8	0.043 (3)	0.022 (2)	0.034 (3)	0.004 (2)	0.015 (2)	0.004 (2)
C9	0.019 (2)	0.0203 (19)	0.026 (2)	0.0082 (17)	0.0030 (19)	0.0099 (18)
C10	0.021 (2)	0.027 (2)	0.028 (2)	0.0086 (18)	0.0032 (19)	0.0094 (18)
C11	0.040 (3)	0.039 (3)	0.031 (3)	0.022 (2)	0.012 (2)	0.015 (2)
C12	0.041 (3)	0.040 (3)	0.031 (3)	0.020 (2)	0.002 (2)	0.010 (2)
C13	0.030 (3)	0.045 (3)	0.041 (3)	0.020 (2)	−0.001 (2)	0.007 (2)
C14	0.026 (2)	0.042 (3)	0.032 (3)	0.017 (2)	0.001 (2)	0.002 (2)
C15	0.017 (2)	0.022 (2)	0.036 (3)	−0.0003 (17)	−0.001 (2)	0.0022 (19)
C16	0.033 (3)	0.031 (3)	0.103 (6)	−0.004 (2)	−0.004 (3)	0.031 (3)
C17	0.042 (3)	0.038 (3)	0.071 (5)	0.005 (2)	−0.003 (3)	0.024 (3)
C18	0.025 (2)	0.027 (2)	0.038 (3)	0.0103 (19)	0.006 (2)	0.010 (2)
C19	0.019 (2)	0.0202 (19)	0.020 (2)	0.0090 (17)	0.0055 (18)	0.0053 (16)
C20	0.024 (2)	0.028 (2)	0.026 (2)	0.0072 (18)	0.006 (2)	0.0041 (18)
C21	0.040 (3)	0.024 (2)	0.051 (3)	0.015 (2)	0.005 (3)	0.012 (2)
C22	0.084 (5)	0.038 (3)	0.065 (5)	0.015 (3)	0.028 (4)	0.026 (3)
C23	0.087 (5)	0.057 (4)	0.105 (6)	0.021 (4)	0.070 (5)	0.030 (4)
C24	0.063 (4)	0.053 (3)	0.046 (4)	0.024 (3)	0.032 (3)	0.015 (3)
C25	0.054 (3)	0.037 (3)	0.037 (3)	0.028 (3)	0.001 (3)	0.014 (2)
C26	0.044 (3)	0.054 (3)	0.047 (3)	0.034 (3)	0.012 (3)	0.021 (3)
C27	0.030 (3)	0.041 (3)	0.041 (3)	0.010 (2)	0.002 (2)	0.014 (2)
C28	0.039 (3)	0.033 (2)	0.035 (3)	0.015 (2)	0.007 (2)	0.013 (2)
C29	0.025 (2)	0.0141 (18)	0.027 (2)	0.0089 (16)	0.0095 (19)	0.0046 (17)
C30	0.032 (2)	0.021 (2)	0.030 (3)	0.0148 (18)	0.012 (2)	0.0074 (18)
C31	0.028 (2)	0.029 (2)	0.034 (3)	0.0170 (19)	0.006 (2)	0.009 (2)
C32	0.034 (3)	0.044 (3)	0.048 (3)	0.025 (2)	0.016 (3)	0.014 (2)
C33	0.042 (3)	0.047 (3)	0.052 (3)	0.027 (2)	0.029 (3)	0.025 (3)
C34	0.029 (2)	0.026 (2)	0.030 (3)	0.0142 (19)	0.013 (2)	0.0135 (19)
C35	0.028 (2)	0.022 (2)	0.022 (2)	0.0101 (18)	−0.0012 (19)	0.0013 (17)
C36	0.033 (3)	0.017 (2)	0.030 (3)	0.0069 (18)	−0.002 (2)	−0.0012 (18)
C37	0.025 (2)	0.0165 (19)	0.033 (3)	0.0086 (17)	0.006 (2)	0.0031 (18)
C38	0.023 (2)	0.0187 (19)	0.026 (2)	0.0115 (17)	0.0050 (19)	0.0076 (17)
C39	0.022 (2)	0.0201 (19)	0.019 (2)	0.0086 (17)	0.0046 (18)	0.0046 (16)
C40	0.027 (2)	0.023 (2)	0.019 (2)	0.0095 (18)	0.0011 (19)	0.0027 (17)
C41	0.031 (2)	0.035 (2)	0.032 (3)	0.024 (2)	0.011 (2)	0.007 (2)
C42	0.036 (3)	0.050 (3)	0.071 (4)	0.025 (3)	0.015 (3)	0.018 (3)
C43	0.041 (3)	0.034 (3)	0.042 (3)	0.022 (2)	0.008 (3)	0.003 (2)
C44A	0.037 (6)	0.031 (5)	0.039 (6)	0.010 (4)	0.006 (5)	−0.013 (4)
C44B	0.031 (4)	0.021 (4)	0.031 (4)	0.016 (3)	0.004 (4)	−0.004 (3)
C45A	0.041 (6)	0.057 (8)	0.044 (7)	0.021 (6)	−0.001 (5)	−0.019 (7)
C46A	0.041 (8)	0.030 (6)	0.029 (7)	0.015 (7)	0.005 (7)	−0.012 (6)
O11	0.0321 (19)	0.0404 (19)	0.051 (2)	0.0164 (15)	0.0136 (18)	0.0127 (17)
C47	0.027 (3)	0.044 (3)	0.055 (4)	0.014 (2)	0.011 (3)	0.017 (3)
C48	0.055 (4)	0.043 (3)	0.065 (4)	0.025 (3)	0.003 (3)	0.010 (3)
C49	0.036 (3)	0.071 (4)	0.057 (4)	0.027 (3)	0.008 (3)	0.011 (3)
C46B	0.045 (9)	0.026 (6)	0.025 (7)	0.007 (7)	0.004 (7)	−0.003 (6)
C45B	0.063 (8)	0.031 (8)	0.048 (9)	−0.004 (6)	0.017 (7)	−0.015 (7)

### Geometric parameters (Å, º)

**Table:**

Nd1—Na1	3.4999 (15)	C12—H12B	0.9900
Nd1—O1	2.364 (3)	C12—C13	1.515 (6)
Nd1—O2	2.422 (3)	C13—H13A	0.9900
Nd1—O3	2.431 (3)	C13—H13B	0.9900
Nd1—O4	2.478 (2)	C13—C14	1.518 (6)
Nd1—O5	2.366 (3)	C14—H14A	0.9900
Nd1—O6	2.573 (2)	C14—H14B	0.9900
Nd1—O7	2.413 (3)	C15—H15A	0.9900
Nd1—O9	2.543 (3)	C15—H15B	0.9900
Cl1—C10	1.764 (5)	C15—C16	1.511 (6)
Cl2—C10	1.765 (4)	C16—H16A	0.9900
Cl3—C10	1.787 (4)	C16—H16B	0.9900
Cl4A—C20	1.7595 (18)	C16—C17	1.454 (8)
Cl4B—C20	1.757 (2)	C17—H17A	0.9900
Cl5A—C20	1.7471 (19)	C17—H17B	0.9900
Cl5B—C20	1.763 (2)	C17—C18	1.514 (6)
Cl6A—C20	1.7583 (18)	C18—H18A	0.9900
Cl6B—C20	1.749 (2)	C18—H18B	0.9900
Cl7—C30	1.782 (4)	C19—C20	1.521 (5)
Cl8—C30	1.763 (5)	C21—H21A	0.9900
Cl9—Na1	3.0192 (19)	C21—H21B	0.9900
Cl9—C30	1.771 (4)	C21—C22	1.515 (7)
Cl10—C40	1.770 (4)	C22—H22A	0.9900
Cl11—C40	1.754 (4)	C22—H22B	0.9900
Cl12—C40	1.761 (4)	C22—C23	1.460 (8)
P1—O1	1.501 (3)	C23—H23A	0.9900
P1—N1	1.630 (3)	C23—H23B	0.9900
P1—N2	1.633 (4)	C23—C24	1.523 (8)
P1—N3	1.633 (3)	C24—H24A	0.9900
P2—O3	1.500 (3)	C24—H24B	0.9900
P2—N4	1.622 (3)	C25—H25A	0.9900
P2—N5	1.623 (4)	C25—H25B	0.9900
P2—N6	1.676 (3)	C25—C26	1.525 (7)
P3—O5	1.500 (3)	C26—H26A	0.9900
P3—N7	1.625 (3)	C26—H26B	0.9900
P3—N8	1.633 (4)	C26—C27	1.519 (7)
P3—N9	1.646 (4)	C27—H27A	0.9900
P4—Na1	3.3404 (17)	C27—H27B	0.9900
P4—O7	1.505 (3)	C27—C28	1.520 (7)
P4—N10	1.633 (3)	C28—H28A	0.9900
P4—N11	1.640 (3)	C28—H28B	0.9900
P4—N12	1.643 (3)	C29—C30	1.563 (5)
Na1—O3	2.407 (3)	C31—H31A	0.9900
Na1—O6	2.369 (3)	C31—H31B	0.9900
Na1—O7	2.418 (3)	C31—C32	1.507 (6)
Na1—O8	2.296 (3)	C32—H32A	0.9900
Na1—O10	2.275 (3)	C32—H32B	0.9900
O2—C9	1.248 (5)	C32—C33	1.525 (7)
O4—C19	1.255 (5)	C33—H33A	0.9900
O6—C29	1.257 (5)	C33—H33B	0.9900
O8—C39	1.240 (4)	C33—C34	1.520 (5)
O9—H9	0.8400	C34—H34A	0.9900
O9—C41	1.441 (4)	C34—H34B	0.9900
O10—H10	0.8576	C35—H35A	0.9900
O10—H10A	0.8530	C35—H35B	0.9900
O10—C44A	1.449 (7)	C35—C36	1.527 (5)
O10—C44B	1.440 (6)	C36—H36A	0.9900
N1—C9	1.298 (5)	C36—H36B	0.9900
N2—C1	1.467 (5)	C36—C37	1.529 (6)
N2—C4	1.472 (6)	C37—H37A	0.9900
N3—C5	1.468 (5)	C37—H37B	0.9900
N3—C8	1.458 (5)	C37—C38	1.516 (5)
N4—C19	1.288 (5)	C38—H38A	0.9900
N5—C11	1.477 (5)	C38—H38B	0.9900
N5—C14	1.475 (5)	C39—C40	1.564 (6)
N6—C15	1.494 (5)	C41—H41	1.0000
N6—C18	1.502 (5)	C41—C42	1.501 (7)
N7—C29	1.292 (5)	C41—C43	1.522 (6)
N8—C21	1.479 (5)	C42—H42A	0.9800
N8—C24	1.472 (6)	C42—H42B	0.9800
N9—C25	1.479 (5)	C42—H42C	0.9800
N9—C28	1.467 (6)	C43—H43A	0.9800
N10—C39	1.325 (5)	C43—H43B	0.9800
N11—C31	1.465 (5)	C43—H43C	0.9800
N11—C34	1.473 (5)	C44A—H44A	1.0000
N12—C35	1.483 (5)	C44A—C46B	1.508 (5)
N12—C38	1.490 (5)	C44A—C45B	1.509 (4)
C1—H1A	0.9900	C44B—H44B	1.0000
C1—H1B	0.9900	C44B—C45A	1.5096 (19)
C1—C2	1.501 (6)	C44B—C46A	1.5096 (19)
C2—H2A	0.9900	C45A—H45A	0.9800
C2—H2B	0.9900	C45A—H45B	0.9800
C2—C3	1.529 (8)	C45A—H45C	0.9800
C3—H3A	0.9900	C46A—H46A	0.9800
C3—H3B	0.9900	C46A—H46B	0.9800
C3—C4	1.549 (7)	C46A—H46C	0.9800
C4—H4A	0.9900	O11—H11	0.8400
C4—H4B	0.9900	O11—C47	1.434 (6)
C5—H5A	0.9900	C47—H47	1.0000
C5—H5B	0.9900	C47—C48	1.518 (7)
C5—C6	1.509 (7)	C47—C49	1.521 (8)
C6—H6A	0.9900	C48—H48A	0.9800
C6—H6B	0.9900	C48—H48B	0.9800
C6—C7	1.515 (8)	C48—H48C	0.9800
C7—H7A	0.9900	C49—H49A	0.9800
C7—H7B	0.9900	C49—H49B	0.9800
C7—C8	1.521 (6)	C49—H49C	0.9800
C8—H8A	0.9900	C46B—H46D	0.9800
C8—H8B	0.9900	C46B—H46E	0.9800
C9—C10	1.567 (5)	C46B—H46F	0.9800
C11—H11A	0.9900	C45B—H45D	0.9800
C11—H11B	0.9900	C45B—H45E	0.9800
C11—C12	1.526 (6)	C45B—H45F	0.9800
C12—H12A	0.9900		
			
O1—Nd1—Na1	156.64 (7)	N6—C15—H15B	111.1
O1—Nd1—O2	73.20 (9)	N6—C15—C16	103.5 (4)
O1—Nd1—O3	147.74 (8)	H15A—C15—H15B	109.0
O1—Nd1—O4	82.23 (9)	C16—C15—H15A	111.1
O1—Nd1—O5	84.29 (9)	C16—C15—H15B	111.1
O1—Nd1—O6	142.69 (9)	C15—C16—H16A	110.5
O1—Nd1—O7	113.26 (8)	C15—C16—H16B	110.5
O1—Nd1—O9	73.00 (9)	H16A—C16—H16B	108.7
O2—Nd1—Na1	93.66 (7)	C17—C16—C15	106.1 (4)
O2—Nd1—O3	136.80 (9)	C17—C16—H16A	110.5
O2—Nd1—O4	146.69 (10)	C17—C16—H16B	110.5
O2—Nd1—O6	74.39 (9)	C16—C17—H17A	110.5
O2—Nd1—O9	117.26 (9)	C16—C17—H17B	110.5
O3—Nd1—Na1	43.39 (6)	C16—C17—C18	106.3 (4)
O3—Nd1—O4	73.55 (9)	H17A—C17—H17B	108.7
O3—Nd1—O6	69.13 (9)	C18—C17—H17A	110.5
O3—Nd1—O9	80.20 (9)	C18—C17—H17B	110.5
O4—Nd1—Na1	116.43 (7)	N6—C18—C17	105.0 (4)
O4—Nd1—O6	117.73 (9)	N6—C18—H18A	110.8
O4—Nd1—O9	74.79 (9)	N6—C18—H18B	110.8
O5—Nd1—Na1	113.00 (7)	C17—C18—H18A	110.8
O5—Nd1—O2	80.20 (10)	C17—C18—H18B	110.8
O5—Nd1—O3	109.02 (9)	H18A—C18—H18B	108.8
O5—Nd1—O4	75.21 (10)	O4—C19—N4	131.8 (4)
O5—Nd1—O6	72.41 (9)	O4—C19—C20	114.8 (3)
O5—Nd1—O7	143.36 (9)	N4—C19—C20	113.4 (3)
O5—Nd1—O9	144.35 (9)	Cl4B—C20—Cl5B	108.0 (5)
O6—Nd1—Na1	42.60 (7)	Cl5A—C20—Cl4A	107.64 (19)
O7—Nd1—Na1	43.62 (6)	Cl5A—C20—Cl6A	106.51 (19)
O7—Nd1—O2	75.18 (9)	Cl6A—C20—Cl4A	105.9 (2)
O7—Nd1—O3	73.76 (9)	Cl6B—C20—Cl4B	112.9 (4)
O7—Nd1—O4	136.53 (9)	Cl6B—C20—Cl5B	97.6 (4)
O7—Nd1—O6	75.07 (8)	C19—C20—Cl4A	112.8 (2)
O7—Nd1—O9	72.08 (8)	C19—C20—Cl4B	110.5 (3)
O9—Nd1—Na1	97.41 (7)	C19—C20—Cl5A	113.8 (2)
O9—Nd1—O6	140.01 (9)	C19—C20—Cl5B	110.8 (3)
C30—Cl9—Na1	97.76 (14)	C19—C20—Cl6A	109.7 (2)
O1—P1—N1	117.19 (17)	C19—C20—Cl6B	116.1 (3)
O1—P1—N2	106.08 (17)	N8—C21—H21A	111.1
O1—P1—N3	114.01 (18)	N8—C21—H21B	111.1
N1—P1—N2	111.06 (19)	N8—C21—C22	103.5 (4)
N1—P1—N3	102.02 (17)	H21A—C21—H21B	109.0
N2—P1—N3	106.06 (19)	C22—C21—H21A	111.1
O3—P2—N4	118.87 (17)	C22—C21—H21B	111.1
O3—P2—N5	112.08 (16)	C21—C22—H22A	110.5
O3—P2—N6	105.45 (15)	C21—C22—H22B	110.5
N4—P2—N5	105.79 (18)	H22A—C22—H22B	108.7
N4—P2—N6	106.22 (17)	C23—C22—C21	106.1 (4)
N5—P2—N6	107.86 (18)	C23—C22—H22A	110.5
O5—P3—N7	116.24 (17)	C23—C22—H22B	110.5
O5—P3—N8	113.11 (18)	C22—C23—H23A	110.6
O5—P3—N9	108.16 (17)	C22—C23—H23B	110.6
N7—P3—N8	103.82 (18)	C22—C23—C24	105.8 (5)
N7—P3—N9	109.02 (19)	H23A—C23—H23B	108.7
N8—P3—N9	105.97 (19)	C24—C23—H23A	110.6
O7—P4—Na1	41.09 (10)	C24—C23—H23B	110.6
O7—P4—N10	117.46 (16)	N8—C24—C23	101.3 (4)
O7—P4—N11	106.67 (15)	N8—C24—H24A	111.5
O7—P4—N12	109.72 (16)	N8—C24—H24B	111.5
N10—P4—Na1	87.36 (11)	C23—C24—H24A	111.5
N10—P4—N11	110.38 (17)	C23—C24—H24B	111.5
N10—P4—N12	102.86 (16)	H24A—C24—H24B	109.3
N11—P4—Na1	94.19 (12)	N9—C25—H25A	110.7
N11—P4—N12	109.61 (17)	N9—C25—H25B	110.7
N12—P4—Na1	148.27 (12)	N9—C25—C26	105.4 (4)
Cl9—Na1—Nd1	107.16 (5)	H25A—C25—H25B	108.8
Cl9—Na1—P4	111.26 (5)	C26—C25—H25A	110.7
P4—Na1—Nd1	67.55 (3)	C26—C25—H25B	110.7
O3—Na1—Nd1	43.94 (7)	C25—C26—H26A	110.8
O3—Na1—Cl9	133.14 (9)	C25—C26—H26B	110.8
O3—Na1—P4	91.64 (7)	H26A—C26—H26B	108.9
O3—Na1—O7	74.11 (9)	C27—C26—C25	104.6 (4)
O6—Na1—Nd1	47.32 (6)	C27—C26—H26A	110.8
O6—Na1—Cl9	63.39 (7)	C27—C26—H26B	110.8
O6—Na1—P4	99.80 (8)	C26—C27—H27A	111.2
O6—Na1—O3	73.01 (10)	C26—C27—H27B	111.2
O6—Na1—O7	78.85 (10)	C26—C27—C28	103.1 (4)
O7—Na1—Nd1	43.52 (7)	H27A—C27—H27B	109.1
O7—Na1—Cl9	111.49 (9)	C28—C27—H27A	111.2
O7—Na1—P4	24.16 (7)	C28—C27—H27B	111.2
O8—Na1—Nd1	124.61 (8)	N9—C28—C27	102.5 (3)
O8—Na1—Cl9	102.95 (9)	N9—C28—H28A	111.3
O8—Na1—P4	58.46 (8)	N9—C28—H28B	111.3
O8—Na1—O3	123.72 (11)	C27—C28—H28A	111.3
O8—Na1—O6	149.95 (12)	C27—C28—H28B	111.3
O8—Na1—O7	82.44 (10)	H28A—C28—H28B	109.2
O10—Na1—Nd1	126.63 (9)	O6—C29—N7	132.1 (4)
O10—Na1—Cl9	92.16 (10)	O6—C29—C30	116.3 (4)
O10—Na1—P4	148.48 (12)	N7—C29—C30	111.6 (3)
O10—Na1—O3	86.74 (11)	Cl8—C30—Cl7	109.5 (2)
O10—Na1—O6	109.72 (12)	Cl8—C30—Cl9	108.0 (2)
O10—Na1—O7	155.97 (12)	Cl9—C30—Cl7	107.8 (2)
O10—Na1—O8	96.86 (13)	C29—C30—Cl7	107.0 (3)
P1—O1—Nd1	132.59 (14)	C29—C30—Cl8	111.3 (3)
C9—O2—Nd1	134.2 (2)	C29—C30—Cl9	113.2 (3)
P2—O3—Nd1	134.08 (15)	N11—C31—H31A	111.2
P2—O3—Na1	133.18 (16)	N11—C31—H31B	111.2
Na1—O3—Nd1	92.67 (9)	N11—C31—C32	103.0 (3)
C19—O4—Nd1	137.0 (3)	H31A—C31—H31B	109.1
P3—O5—Nd1	138.48 (17)	C32—C31—H31A	111.2
Na1—O6—Nd1	90.08 (9)	C32—C31—H31B	111.2
C29—O6—Nd1	133.7 (3)	C31—C32—H32A	111.2
C29—O6—Na1	135.6 (3)	C31—C32—H32B	111.2
Nd1—O7—Na1	92.86 (9)	C31—C32—C33	102.7 (4)
P4—O7—Nd1	151.59 (15)	H32A—C32—H32B	109.1
P4—O7—Na1	114.75 (15)	C33—C32—H32A	111.2
C39—O8—Na1	126.2 (2)	C33—C32—H32B	111.2
Nd1—O9—H9	120.2	C32—C33—H33A	110.9
C41—O9—Nd1	127.9 (3)	C32—C33—H33B	110.9
C41—O9—H9	109.5	H33A—C33—H33B	108.9
Na1—O10—H10	122.2	C34—C33—C32	104.5 (3)
Na1—O10—H10A	110.4	C34—C33—H33A	110.9
C44A—O10—Na1	133.5 (4)	C34—C33—H33B	110.9
C44A—O10—H10	101.2	N11—C34—C33	104.8 (3)
C44B—O10—Na1	138.6 (3)	N11—C34—H34A	110.8
C44B—O10—H10A	110.3	N11—C34—H34B	110.8
C9—N1—P1	122.0 (3)	C33—C34—H34A	110.8
C1—N2—P1	125.3 (3)	C33—C34—H34B	110.8
C1—N2—C4	111.4 (4)	H34A—C34—H34B	108.9
C4—N2—P1	123.0 (3)	N12—C35—H35A	110.8
C5—N3—P1	119.5 (3)	N12—C35—H35B	110.8
C8—N3—P1	128.4 (3)	N12—C35—C36	104.9 (3)
C8—N3—C5	112.1 (3)	H35A—C35—H35B	108.8
C19—N4—P2	124.3 (3)	C36—C35—H35A	110.8
C11—N5—P2	121.2 (3)	C36—C35—H35B	110.8
C14—N5—P2	126.7 (3)	C35—C36—H36A	111.0
C14—N5—C11	111.8 (3)	C35—C36—H36B	111.0
C15—N6—P2	116.9 (3)	C35—C36—C37	103.6 (3)
C15—N6—C18	108.6 (3)	H36A—C36—H36B	109.0
C18—N6—P2	114.1 (3)	C37—C36—H36A	111.0
C29—N7—P3	124.6 (3)	C37—C36—H36B	111.0
C21—N8—P3	125.7 (3)	C36—C37—H37A	111.3
C24—N8—P3	120.4 (3)	C36—C37—H37B	111.3
C24—N8—C21	111.7 (4)	H37A—C37—H37B	109.2
C25—N9—P3	119.0 (3)	C38—C37—C36	102.2 (3)
C28—N9—P3	121.1 (3)	C38—C37—H37A	111.3
C28—N9—C25	109.2 (4)	C38—C37—H37B	111.3
C39—N10—P4	115.8 (3)	N12—C38—C37	103.7 (3)
C31—N11—P4	124.6 (3)	N12—C38—H38A	111.0
C31—N11—C34	110.0 (3)	N12—C38—H38B	111.0
C34—N11—P4	121.7 (3)	C37—C38—H38A	111.0
C35—N12—P4	117.3 (2)	C37—C38—H38B	111.0
C35—N12—C38	109.3 (3)	H38A—C38—H38B	109.0
C38—N12—P4	123.2 (3)	O8—C39—N10	130.1 (4)
N2—C1—H1A	111.1	O8—C39—C40	114.2 (3)
N2—C1—H1B	111.1	N10—C39—C40	115.7 (3)
N2—C1—C2	103.5 (4)	Cl11—C40—Cl10	108.5 (2)
H1A—C1—H1B	109.0	Cl11—C40—Cl12	108.9 (2)
C2—C1—H1A	111.1	Cl12—C40—Cl10	108.2 (2)
C2—C1—H1B	111.1	C39—C40—Cl10	106.7 (3)
C1—C2—H2A	111.4	C39—C40—Cl11	114.1 (3)
C1—C2—H2B	111.4	C39—C40—Cl12	110.3 (3)
C1—C2—C3	102.0 (4)	O9—C41—H41	108.0
H2A—C2—H2B	109.2	O9—C41—C42	110.1 (4)
C3—C2—H2A	111.4	O9—C41—C43	109.6 (3)
C3—C2—H2B	111.4	C42—C41—H41	108.0
C2—C3—H3A	111.3	C42—C41—C43	113.0 (4)
C2—C3—H3B	111.3	C43—C41—H41	108.0
C2—C3—C4	102.4 (4)	C41—C42—H42A	109.5
H3A—C3—H3B	109.2	C41—C42—H42B	109.5
C4—C3—H3A	111.3	C41—C42—H42C	109.5
C4—C3—H3B	111.3	H42A—C42—H42B	109.5
N2—C4—C3	102.4 (4)	H42A—C42—H42C	109.5
N2—C4—H4A	111.3	H42B—C42—H42C	109.5
N2—C4—H4B	111.3	C41—C43—H43A	109.5
C3—C4—H4A	111.3	C41—C43—H43B	109.5
C3—C4—H4B	111.3	C41—C43—H43C	109.5
H4A—C4—H4B	109.2	H43A—C43—H43B	109.5
N3—C5—H5A	111.1	H43A—C43—H43C	109.5
N3—C5—H5B	111.1	H43B—C43—H43C	109.5
N3—C5—C6	103.1 (4)	O10—C44A—H44A	107.5
H5A—C5—H5B	109.1	O10—C44A—C46B	109.1 (10)
C6—C5—H5A	111.1	O10—C44A—C45B	112.8 (10)
C6—C5—H5B	111.1	C46B—C44A—H44A	107.5
C5—C6—H6A	111.1	C46B—C44A—C45B	112.3 (14)
C5—C6—H6B	111.1	C45B—C44A—H44A	107.5
C5—C6—C7	103.3 (4)	O10—C44B—H44B	112.0
H6A—C6—H6B	109.1	O10—C44B—C45A	103.2 (8)
C7—C6—H6A	111.1	O10—C44B—C46A	104.9 (8)
C7—C6—H6B	111.1	C45A—C44B—H44B	112.0
C6—C7—H7A	111.2	C45A—C44B—C46A	112.3 (11)
C6—C7—H7B	111.2	C46A—C44B—H44B	112.0
C6—C7—C8	102.7 (4)	C44B—C45A—H45A	109.5
H7A—C7—H7B	109.1	C44B—C45A—H45B	109.5
C8—C7—H7A	111.2	C44B—C45A—H45C	109.5
C8—C7—H7B	111.2	H45A—C45A—H45B	109.5
N3—C8—C7	102.3 (4)	H45A—C45A—H45C	109.5
N3—C8—H8A	111.3	H45B—C45A—H45C	109.5
N3—C8—H8B	111.3	C44B—C46A—H46A	109.5
C7—C8—H8A	111.3	C44B—C46A—H46B	109.5
C7—C8—H8B	111.3	C44B—C46A—H46C	109.5
H8A—C8—H8B	109.2	H46A—C46A—H46B	109.5
O2—C9—N1	133.0 (4)	H46A—C46A—H46C	109.5
O2—C9—C10	113.1 (3)	H46B—C46A—H46C	109.5
N1—C9—C10	113.8 (4)	C47—O11—H11	109.5
Cl1—C10—Cl2	108.8 (2)	O11—C47—H47	109.2
Cl1—C10—Cl3	108.0 (2)	O11—C47—C48	107.6 (4)
Cl2—C10—Cl3	108.8 (2)	O11—C47—C49	111.1 (4)
C9—C10—Cl1	113.6 (3)	C48—C47—H47	109.2
C9—C10—Cl2	111.3 (3)	C48—C47—C49	110.4 (4)
C9—C10—Cl3	106.2 (3)	C49—C47—H47	109.2
N5—C11—H11A	111.1	C47—C48—H48A	109.5
N5—C11—H11B	111.1	C47—C48—H48B	109.5
N5—C11—C12	103.2 (3)	C47—C48—H48C	109.5
H11A—C11—H11B	109.1	H48A—C48—H48B	109.5
C12—C11—H11A	111.1	H48A—C48—H48C	109.5
C12—C11—H11B	111.1	H48B—C48—H48C	109.5
C11—C12—H12A	111.0	C47—C49—H49A	109.5
C11—C12—H12B	111.0	C47—C49—H49B	109.5
H12A—C12—H12B	109.0	C47—C49—H49C	109.5
C13—C12—C11	103.6 (4)	H49A—C49—H49B	109.5
C13—C12—H12A	111.0	H49A—C49—H49C	109.5
C13—C12—H12B	111.0	H49B—C49—H49C	109.5
C12—C13—H13A	111.0	C44A—C46B—H46D	109.5
C12—C13—H13B	111.0	C44A—C46B—H46E	109.5
C12—C13—C14	103.8 (4)	C44A—C46B—H46F	109.5
H13A—C13—H13B	109.0	H46D—C46B—H46E	109.5
C14—C13—H13A	111.0	H46D—C46B—H46F	109.5
C14—C13—H13B	111.0	H46E—C46B—H46F	109.5
N5—C14—C13	102.8 (3)	C44A—C45B—H45D	109.5
N5—C14—H14A	111.2	C44A—C45B—H45E	109.5
N5—C14—H14B	111.2	C44A—C45B—H45F	109.5
C13—C14—H14A	111.2	H45D—C45B—H45E	109.5
C13—C14—H14B	111.2	H45D—C45B—H45F	109.5
H14A—C14—H14B	109.1	H45E—C45B—H45F	109.5
N6—C15—H15A	111.1		
			
Nd1—O2—C9—N1	−16.5 (7)	N3—P1—N2—C1	130.4 (3)
Nd1—O2—C9—C10	165.6 (2)	N3—P1—N2—C4	−56.1 (4)
Nd1—O4—C19—N4	−3.4 (7)	N3—C5—C6—C7	−30.6 (5)
Nd1—O4—C19—C20	178.0 (2)	N4—P2—O3—Nd1	−8.2 (3)
Nd1—O6—C29—N7	13.5 (7)	N4—P2—O3—Na1	168.09 (19)
Nd1—O6—C29—C30	−169.2 (2)	N4—P2—N5—C11	157.9 (3)
Nd1—O9—C41—C42	−125.3 (4)	N4—P2—N5—C14	−15.3 (4)
Nd1—O9—C41—C43	109.8 (4)	N4—P2—N6—C15	59.6 (4)
P1—N1—C9—O2	−3.9 (6)	N4—P2—N6—C18	−68.7 (3)
P1—N1—C9—C10	174.1 (3)	N4—C19—C20—Cl4A	31.4 (4)
P1—N2—C1—C2	156.0 (3)	N4—C19—C20—Cl4B	100.3 (5)
P1—N2—C4—C3	177.3 (3)	N4—C19—C20—Cl5A	154.4 (3)
P1—N3—C5—C6	−170.4 (4)	N4—C19—C20—Cl5B	−140.0 (5)
P1—N3—C8—C7	−164.5 (4)	N4—C19—C20—Cl6A	−86.4 (3)
P2—N4—C19—O4	2.8 (7)	N4—C19—C20—Cl6B	−29.9 (5)
P2—N4—C19—C20	−178.7 (2)	N5—P2—O3—Nd1	115.8 (2)
P2—N5—C11—C12	176.7 (3)	N5—P2—O3—Na1	−67.9 (2)
P2—N5—C14—C13	159.0 (3)	N5—P2—N4—C19	−124.1 (4)
P2—N6—C15—C16	−150.1 (4)	N5—P2—N6—C15	−53.4 (3)
P2—N6—C18—C17	133.0 (4)	N5—P2—N6—C18	178.3 (3)
P3—N7—C29—O6	0.7 (7)	N5—C11—C12—C13	29.4 (5)
P3—N7—C29—C30	−176.7 (3)	N6—P2—O3—Nd1	−127.1 (2)
P3—N8—C21—C22	165.0 (4)	N6—P2—O3—Na1	49.2 (3)
P3—N8—C24—C23	174.8 (4)	N6—P2—N4—C19	121.4 (4)
P3—N9—C25—C26	154.8 (3)	N6—P2—N5—C11	−88.8 (3)
P3—N9—C28—C27	−174.7 (3)	N6—P2—N5—C14	98.0 (4)
P4—N10—C39—O8	15.7 (5)	N6—C15—C16—C17	31.4 (6)
P4—N10—C39—C40	−162.1 (3)	N7—P3—O5—Nd1	16.8 (4)
P4—N11—C31—C32	−174.1 (3)	N7—P3—N8—C21	15.6 (4)
P4—N11—C34—C33	−164.6 (3)	N7—P3—N8—C24	177.1 (4)
P4—N12—C35—C36	149.7 (3)	N7—P3—N9—C25	−59.5 (4)
P4—N12—C38—C37	−122.6 (3)	N7—P3—N9—C28	81.1 (3)
Na1—Cl9—C30—Cl7	87.16 (18)	N7—C29—C30—Cl7	75.2 (4)
Na1—Cl9—C30—Cl8	−154.65 (17)	N7—C29—C30—Cl8	−44.4 (4)
Na1—Cl9—C30—C29	−31.0 (3)	N7—C29—C30—Cl9	−166.2 (3)
Na1—P4—O7—Nd1	−165.3 (4)	N8—P3—O5—Nd1	−103.2 (3)
Na1—P4—N10—C39	−33.8 (3)	N8—P3—N7—C29	110.1 (4)
Na1—P4—N11—C31	−24.5 (4)	N8—P3—N9—C25	51.7 (4)
Na1—P4—N11—C34	131.5 (3)	N8—P3—N9—C28	−167.7 (3)
Na1—P4—N12—C35	80.1 (4)	N8—C21—C22—C23	19.2 (7)
Na1—P4—N12—C38	−138.4 (2)	N9—P3—O5—Nd1	139.7 (3)
Na1—O6—C29—N7	−154.2 (4)	N9—P3—N7—C29	−137.3 (4)
Na1—O6—C29—C30	23.1 (5)	N9—P3—N8—C21	−99.2 (4)
Na1—O8—C39—N10	36.4 (6)	N9—P3—N8—C24	62.3 (4)
Na1—O8—C39—C40	−145.8 (3)	N9—C25—C26—C27	15.1 (5)
Na1—O10—C44A—C46B	53.1 (14)	N10—P4—O7—Nd1	−117.8 (3)
Na1—O10—C44A—C45B	178.6 (10)	N10—P4—O7—Na1	47.6 (2)
Na1—O10—C44B—C45A	−136.7 (8)	N10—P4—N11—C31	−113.2 (4)
Na1—O10—C44B—C46A	−18.9 (12)	N10—P4—N11—C34	42.8 (4)
O1—P1—N1—C9	−0.5 (4)	N10—P4—N12—C35	−173.7 (3)
O1—P1—N2—C1	8.8 (4)	N10—P4—N12—C38	−32.2 (3)
O1—P1—N2—C4	−177.6 (4)	N10—C39—C40—Cl10	112.7 (3)
O1—P1—N3—C5	60.5 (4)	N10—C39—C40—Cl11	−7.1 (4)
O1—P1—N3—C8	−118.8 (4)	N10—C39—C40—Cl12	−130.1 (3)
O2—C9—C10—Cl1	−156.1 (3)	N11—P4—O7—Nd1	117.8 (3)
O2—C9—C10—Cl2	−32.9 (4)	N11—P4—O7—Na1	−76.86 (19)
O2—C9—C10—Cl3	85.3 (4)	N11—P4—N10—C39	59.7 (3)
O3—P2—N4—C19	2.9 (4)	N11—P4—N12—C35	−56.2 (3)
O3—P2—N5—C11	26.8 (4)	N11—P4—N12—C38	85.2 (3)
O3—P2—N5—C14	−146.3 (3)	N11—C31—C32—C33	−38.0 (5)
O3—P2—N6—C15	−173.4 (3)	N12—P4—O7—Nd1	−0.9 (4)
O3—P2—N6—C18	58.3 (3)	N12—P4—O7—Na1	164.49 (15)
O4—C19—C20—Cl4A	−149.8 (3)	N12—P4—N10—C39	176.6 (3)
O4—C19—C20—Cl4B	−80.8 (5)	N12—P4—N11—C31	134.2 (3)
O4—C19—C20—Cl5A	−26.8 (4)	N12—P4—N11—C34	−69.9 (4)
O4—C19—C20—Cl5B	38.8 (6)	N12—C35—C36—C37	−26.5 (4)
O4—C19—C20—Cl6A	92.5 (3)	C1—N2—C4—C3	−8.4 (5)
O4—C19—C20—Cl6B	148.9 (4)	C1—C2—C3—C4	−42.5 (5)
O5—P3—N7—C29	−14.8 (4)	C2—C3—C4—N2	31.1 (5)
O5—P3—N8—C21	142.5 (4)	C4—N2—C1—C2	−18.2 (5)
O5—P3—N8—C24	−56.1 (5)	C5—N3—C8—C7	16.2 (6)
O5—P3—N9—C25	173.3 (3)	C5—C6—C7—C8	40.9 (5)
O5—P3—N9—C28	−46.1 (4)	C6—C7—C8—N3	−34.6 (5)
O6—C29—C30—Cl7	−102.6 (3)	C8—N3—C5—C6	9.0 (6)
O6—C29—C30—Cl8	137.8 (3)	C11—N5—C14—C13	−14.7 (5)
O6—C29—C30—Cl9	16.0 (5)	C11—C12—C13—C14	−39.0 (5)
O7—P4—N10—C39	−62.8 (3)	C12—C13—C14—N5	32.8 (5)
O7—P4—N11—C31	15.5 (4)	C14—N5—C11—C12	−9.2 (5)
O7—P4—N11—C34	171.4 (3)	C15—N6—C18—C17	0.6 (5)
O7—P4—N12—C35	60.6 (3)	C15—C16—C17—C18	−31.8 (7)
O7—P4—N12—C38	−158.0 (3)	C16—C17—C18—N6	19.2 (6)
O8—C39—C40—Cl10	−65.4 (4)	C18—N6—C15—C16	−19.2 (5)
O8—C39—C40—Cl11	174.7 (3)	C21—N8—C24—C23	−21.4 (6)
O8—C39—C40—Cl12	51.8 (4)	C21—C22—C23—C24	−33.2 (8)
N1—P1—O1—Nd1	25.2 (3)	C22—C23—C24—N8	33.0 (7)
N1—P1—N2—C1	−119.5 (3)	C24—N8—C21—C22	2.2 (6)
N1—P1—N2—C4	54.0 (4)	C25—N9—C28—C27	−30.7 (5)
N1—P1—N3—C5	−172.2 (4)	C25—C26—C27—C28	−33.4 (5)
N1—P1—N3—C8	8.5 (5)	C26—C27—C28—N9	39.1 (5)
N1—C9—C10—Cl1	25.6 (4)	C28—N9—C25—C26	9.9 (5)
N1—C9—C10—Cl2	148.8 (3)	C31—N11—C34—C33	−5.5 (5)
N1—C9—C10—Cl3	−93.0 (4)	C31—C32—C33—C34	35.1 (5)
N2—P1—O1—Nd1	−99.5 (2)	C32—C33—C34—N11	−18.5 (5)
N2—P1—N1—C9	121.6 (3)	C34—N11—C31—C32	27.6 (5)
N2—P1—N3—C5	−55.9 (4)	C35—N12—C38—C37	21.5 (4)
N2—P1—N3—C8	124.8 (4)	C35—C36—C37—C38	39.4 (4)
N2—C1—C2—C3	37.2 (4)	C36—C37—C38—N12	−37.3 (4)
N3—P1—O1—Nd1	144.21 (19)	C38—N12—C35—C36	3.2 (4)
N3—P1—N1—C9	−125.7 (3)		

### Hydrogen-bond geometry (Å, º)

**Table:**

*D*—H···*A*	*D*—H	H···*A*	*D*···*A*	*D*—H···*A*
O10—H10*A*···N6	0.85	2.11	2.872 (4)	148
O11—H11···N10	0.84	2.22	3.024 (6)	160
C49—H49*A*···Cl11	0.98	2.82	3.637 (7)	141
